# Factors associated with outcomes of patients on extracorporeal membrane oxygenation support: a 5-year cohort study

**DOI:** 10.1186/cc12681

**Published:** 2013-04-18

**Authors:** Cecile Aubron, Allen C Cheng, David Pilcher, Tim Leong, Geoff Magrin, D Jamie Cooper, Carlos Scheinkestel, Vince Pellegrino

**Affiliations:** 1 The Intensive Care Unit, Alfred Hospital, 55 Commercial Road, Melbourne 3004, VIC, Australia; 2 The Infectious Diseases Unit, Alfred Hospital, 55 Commercial Road, Melbourne 3004, VIC, Australia; 3 The Transfusion Service, Alfred Hospital, 55 Commercial Road, Melbourne 3004, VIC, Australia; 4 The Australian and New Zealand Intensive care Research Centre, Department of Epidemiology and Preventive Medicine, School of Public Health and Preventive Medicine, Monash University, The Alfred Centre, 99 Commercial Road, Melbourne 3004, VIC, Australia; 5 Department of Epidemiology and Preventive Medicine, School of Public Health and Preventive Medicine, Monash University, The Alfred Centre, 99 Commercial Road, Melbourne 3004, VIC, Australia

## Abstract

**Introduction:**

Mortality of patients on extracorporeal membrane oxygenation (ECMO) remains high. The objectives of this study were to assess the factors associated with outcome of patients undergoing ECMO in a large ECMO referral centre and to compare veno-arterial ECMO (VA ECMO) with veno-venous ECMO (VV ECMO).

**Methods:**

We reviewed a prospectively obtained ECMO database and patients' medical records between January 2005 and June 2011. Demographic characteristics, illness severity at admission, ECMO indication, organ failure scores before ECMO and the ECMO mode and configuration were recorded. Bleeding, neurological, vascular and infectious complications that occurred on ECMO were also collected. Demographic, illness, ECMO support descriptors and complications associated with hospital mortality were analysed.

**Results:**

ECMO was initiated 158 times in 151 patients. VA ECMO (66.5%) was twice as common as VV ECMO (33.5%) with a median duration significantly shorter than for VV ECMO (7 days (first and third quartiles: 5; 10 days) versus 10 days (first and third quartiles: 6; 16 days)). The most frequent complications during ECMO support were bleeding and bloodstream infections regardless of ECMO type. More than 70% of the ECMO episodes were successfully weaned in each ECMO group. The overall mortality was 37.3% (37.1% for the patients who underwent VA ECMO, and 37.7% for the patients who underwent VV ECMO). Haemorrhagic events, assessed by the total of red blood cell units received during ECMO, were associated with hospital mortality for both ECMO types.

**Conclusions:**

Among neurologic, vascular, infectious and bleeding events that occurred on ECMO, bleeding was the most frequent and had a significant impact on mortality. Further studies are needed to better investigate bleeding and coagulopathy in these patients. Interventions that reduce these complications may improve outcome.

## Introduction

Extracorporeal membrane oxygenation (ECMO) is a rescue therapy to support severe cardiac and/or pulmonary failure. Both veno-venous (VV) and veno-arterial (VA) ECMO (or extracorporeal life support (ECSL)) are increasingly being used [1-6]. VV ECMO support for severe influenza A (H1N1) pneumonitis was reported during the 2009 pandemic [3,7-9]. Despite increasing experience with ECMO and recent technical improvements, the mortality of patients receiving ECMO remains high, but varies between centres, patient subgroups and by indication [2,4,10-12]. While more randomised controlled trials are needed to define the place of ECMO in critically ill patient management, observational series may provide some useful data on factors associated with mortality and complications.

Outcome of patients on ECMO is influenced not only by factors independent of ECMO (patient illness severity, type of illness, other organ support) but also by the potential complications related to ECMO. Clarification of the impact of key ECMO complications on outcome could inform safer care and improve outcomes. ECMO complications may be mechanical (relating to the ECMO circuit components) or medical [9]. The latter are the most frequent and include bleeding, infection, embolism causing vascular and neurological complications, and limb ischaemia. Haemorrhage and infection are the most frequent adverse events [2,9,13-20]. Nonetheless, neurologic complications are probably underestimated and can have devastating consequences on the prognosis [3,21]. Vascular complications such as amputation may be delayed and also perhaps under-reported. Bloodstream infections during ECMO have been associated with a poor outcome in paediatric patients [17,22] but the association remains uncertain in adults [15,19,23]. Bleeding is another frequent adverse event in these patients who are critical ill, exposed to anticoagulation and susceptible to coagulopathy and platelet dysfunction. The impact of bleeding on prognosis depends in part on how bleeding events are defined and recorded in the studies [2,9,24]. Mechanical complications and haemolysis have decreased with the introduction of centrifugal pumps, low-resistance polymethylpentene membranes and modern heparin-coated surfaces.

This study describes the experience of a single ECMO referral centre during a stable period of practice. The aim of this study was also to identify factors that were independently associated with outcome for VV and VA ECMO.

## Methods

The Alfred Hospital is a university referral hospital in Melbourne (Australia), which provides heart and lung transplantation services for the states of Victoria, South Australia and Tasmania. It is one of two adult trauma centres in Victoria and is the state Burns Centre. The intensive care unit (ICU) is one of the largest Australian ICUs with capacity for 45 beds and more than 2,000 admissions a year. The Alfred ICU operates an ECMO referral service and retrieves patients on ECMO from the southern Australian states.

Data were retrospectively extracted from a prospectively updated local registry of ECMO patients and ICU clinical database. Further clinical details were obtained from retrospective review of patient medical records. This study was approved by the Alfred Health Human Research Ethics Committee and no consent was needed.

### Patients

Patients over the age of 16 years who received ECMO support between January 2005 and June 2011 were included. Demographics, co-morbidities, hospital and ICU lengths of stay, acute physiology and chronic health evaluation (APACHE II) score and main diagnosis at admission were recorded. The following parameters were collected at ECMO initiation: presence of cardiac arrest, sequential organ failure assessment (SOFA) score [25], plasma lactate level and arterial partial pressure of oxygen to inspired oxygen fraction (PaO_2_/FiO_2_) ratio. Days in ICU and days on mechanical ventilation (MV) before ECMO, days on ECMO, and the requirement for catecholamines and renal replacement therapy (RRT) were recorded.

### Patient selection, ECMO configurations and routine care

The decision to use ECMO was made by the treating intensive care specialist or cardiac surgeon (for intra-operative cardiac support). Criteria for assessing the need to commence VV ECMO for severe respiratory failure and VA ECMO for severe cardiac failure are listed in Table 1. For respiratory failure, VV ECMO was delivered through percutaneously placed single-stage femoro-femoral cannulae (Medtronic, Minneapolis, MN, USA) under ultrasound guidance with an additional jugular access cannula inserted if required. Double lumen jugular cannulae (Avalon Elite™; Avalon Laboratories, Rancho Dominguez, CA, USA) were used in two patients. Mechanical ventilation settings were not standardised in patients undergoing MV. Our practice generally involved pressure-controlled ventilation with low respiratory rate and maintenance of tidal volume below 6 ml/kg (predicted body weight) and peak pressure below 30 cm H_2_O. Percutaneous VA ECMO for cardiac support was delivered through peripheral femoro-femoral ECMO cannulae and routinely included an ante-grade 8.5 French distal perfusion cannula (Mayo, Rochester, MN, USA) to prevent limb ischaemia. Central VA ECMO was initiated intra-operatively at the discretion of the managing cardiac surgeon. Management of failing heart was not standardised but low doses of inotropes were administered to maintain pulsatility and some native cardiac output, if these were severely depressed on support. Femoral artery cannulation sites were repaired surgically after decannulation. Continuous renal replacement (Prisma™ and then Prismaflex™; Gambro, Lund, Sweden) was performed via the ECMO circuit. ECMO care was delivered using Rotaflow pumps and Quadrox or PLS membranes with simplified Bioline-coated circuits (Maquet, Rastatt, Germany) without connectors, bridges or venous saturation monitoring. To avoid air emboli, we did not monitor pre-pump pressure and there were no pre-pump circuit access ports. Bedside care was delivered by trained ICU nursing staff with a patient-nurse ratio of 1:1 under the supervision of ECMO-trained intensive care specialists.

**Table 1:** Criteria for the use of extracorporeal membrane oxygenation (ECMO).

Type of organ failure	Criteria
Respiratory failure	1- Treatable underlying respiratory condition 2- Absence of contraindications • *Severe chronic liver disease* • *Severe brain injury* • *Non-responsive malignancy* 3- Requirements for unsafe ventilation to achieve SaO_2 _> 88% or pH > 7.20 • *Plateau pressure > 35 cmH_2_O* • *Tidal volume > 6 ml/Kg predicted body weight (PBW)* 4- With hypoxaemia (SaO2 < 88%) despite • *FiO2 ≥ 90%* • *Trial of high positive end-expiratory pressure (between 18 and 22 cmH_2_O)* • *Trial of recruitment manoeuvre (if not contraindicated)* • *2-12 hour trial of inhaled nitric oxide (NO) if available* • *Adequate cardiac support (echocardiography assessment, inotropes, pulmonary vasodilators)* 5- Or requirement for inter-hospital transport 6- Rate of lung injury progression*
Cardiac failure	1. Diagnosis of cardiogenic shock: • *Echocardiography examination to confirm the presence and nature of cardiac dysfunction and exclude correctible problems* 2. Cardiac index and blood pressure inadequate for organ support despite • *Moderate- or high-dose inotropes (adrenaline > 0.3 μg/Kg/min equivalent) in combination with an intra-aortic balloon pump (IABP), vasopressors and positive pressure ventilation for predominately left ventricular failure* • *Moderate- or high-dose inotropes (adrenaline > 0.3 μg/Kg/min equivalent) in combination with pulmonary artery vasodilator and/or vasopressors for predominately right ventricular failure* 3. Inadequate organ support despite medical therapy as evidenced by • *Onset of hepatic (acute transaminitis), renal (anuria or rising creatinine) dysfunction or skin hypoperfusion (mottled or purpuric)* • *Lactate > 4 mmol/L* 4. Malignant arrhythmia: refractory ventricular fibrillation or tachycardia not otherwise controlled

*Rapidly progressive (6 to 12 hours) lung infiltrates and increasing ventilator requirements particularly in the early stages of hospital admission are often associated with a fulminate illness that reduces the time window when ECMO may be of benefit.

### Anticoagulation, management of bleeding and circuit changes

Invasive procedures were minimised while on ECMO. Platelet transfusions were given to maintain platelet count > 80,000/mm^3^. Minimum haemoglobin of 80 g/l was generally targeted, consistent with reported literature [26]. Patients with no active bleeding received heparin targeted to an activated partial thromboplastin time (APTT) of 50 to 70 seconds. Heparin was withheld at the discretion of the intensivist in patients who required surgical procedures or in those who had clinically significant bleeding. Administration of clotting factors, platelets, antifibrinolytics or recombinant factor VIIa for bleeding patients was determined by the intensivist in consultation with a specialist haematologist. Pre and post oxygenator pressures were monitored to detect progressive oxygenator thrombosis. Screening for haemolysis (plasma-free haemoglobin) and fibrinolysis (fibrinogen and D-dimer levels) was performed at least daily. Elective circuit changes were performed if significant trends in fibrinogen decline and D-dimer elevation were observed.

### Antibiotic management and microbiological samples

Antibiotic prophylaxis for ECMO was not routine. Patients undergoing open heart surgery were given prophylaxis with cefazolin plus rifampicin or vancomycin plus rifampicin in the operating room in accordance with hospital guidelines. Suspected and/or confirmed sepsis was routinely treated with antibiotics following specimens being taken for culture.

### Complications

The following complications were recorded: i) haemorrhagic and coagulopathy: surgical interventions for bleeding (recorded prospectively), transfusion requirement: total red blood cell (RBC) units, total of platelets bags, fresh frozen plasma (FFP) and cryoprecipitate issued during ECMO at the Alfred hospital (recorded retrospectively); ii) bloodstream infections: defined using the Centre for Disease Control and Prevention/National Nosocomial Infections Surveillance System criteria [27] and occurring between 48 hours after ECMO initiation and 72 hours after ECMO cessation (recorded retrospectively); iii) neurologic: defined as a cerebral haemorrhage or ischaemia reported on CT scan with no other potential aetiology; iv) vascular: vascular repair, fasciotomy, embolectomy or a limb amputation during or after ECMO (recorded prospectively).

### Outcomes

Primary outcomes considered were the proportion of patients successfully weaned from ECMO without bridging support and hospital mortality. When they occurred, deaths were classified as related to the primary illness, ECMO care, or occurring subsequently after weaning from ECMO. Hospital and ICU length of stay (LOS), ICU mortality and the percentage of survivors discharged home after hospital discharge were also recorded.

### Statistical analysis

All analyses were performed using Stata 10.0 (College Station, TX, USA). VV and VA ECMO were analysed separately. However, changes over the study period considered all the ECMO episodes in the same analysis. When ECMO was initiated in another centre, only the days at the Alfred hospital were taken into account for the number of RBC units given per day on ECMO. When the mode of ECMO was changed, only the first type was considered for analysis. Categorical variables were compared between groups with a Fisher exact test and continuous variables were compared with a Mann-Whitney *U *test for non-parametric data. Statistical regression models were constructed based on factors found to be associated with hospital mortality using backwards stepwise regression based on *P *< 0.02 or factors considered relevant. In the mortality models, data available at the time of starting ECMO were considered in a prognostic model and the effect of complications was considered in a complications model. To avoid interaction between days on support and number of RBC units per day, the number of RBC units transfused has been considered when applicable. When variables were expected to be collinear (for example SOFA score and APACHE II score), only one was included in the model. A two-sided *P *value of 0.05 was considered to be statistically significant.

## Results

### Patient characteristics

**Figure 1:**
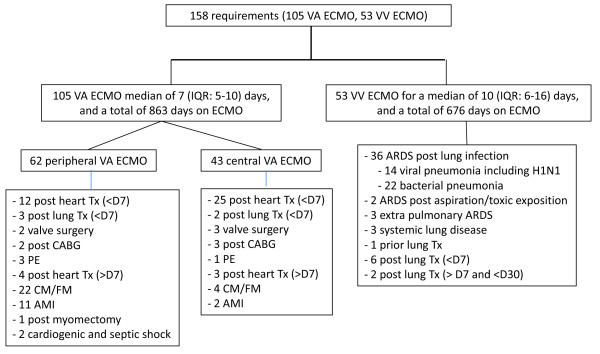
**Primary indications of the 158 extracorporeal membrane oxygenation (ECMO) procedures**. AMI, acute myocardial infarction; ARDS, acute respiratory distress syndrome; CABG, coronary artery bypass surgery; CM/FM, cardiomyopathy/myocarditis; PE, pulmonary emboli; Tx, transplantation.

Between January 2005 and June 2011, there were 9,350 adult admissions to ICU; 151 (1.7%) patients were hospitalised and underwent ECMO support on 158 occasions. Ninety-nine patients underwent 105 VA ECMO for a median duration of 7 days (first and third quartiles: 5 to 10 days), while 52 patients underwent 53 episodes of VV ECMO for a median duration of 10 days (first and third quartiles: 6 to 16 days). The primary indications for ECMO support according to the type of ECMO are described in Figure 1. Forty patients were transferred to the hospital on ECMO, they were mostly on VV ECMO (41% versus 22%, *P *= 0.002), and in 34 (85%) patients, ECMO was initiated less than 24 hours prior to arrival. Two patients changed modes from VA to VV ECMO. Patients and ECMO characteristics are detailed per type of ECMO in Table 2. In the VA ECMO group, the SOFA score before ECMO initiation was higher in patients with peripheral ECMO than in those with central ECMO (12 (first and third quartiles: 11 to 14) versus 11 (first and third quartiles: 10 to 12.5), *P *= 0.0225).

**Table 2:** Patients and extracorporeal membrane oxygenation **(**ECMO) characteristics and comparison between veno-venous (VV) ECMO and veno-arterial (VA) ECMO.

Variables	VA ECMO (*n *= 105)	VV ECMO (*n *= 53)	*P *value for VV versus VA ECMO
Age (years)	50 (43-59)	35 (24-49)	< 0.0001
Female	23 (22%)	20 (37.7%)	0.039
APACHE II score (*n *= 144)	21.5 (16-27)	16.5 (13-21)	0.001
ECMO initiated in another hospital	19 (18%)	22 (41%)	0.002
ECMO post surgery	54 (51%)	6 (11%)	< 0.0001
Days on support	7 (5-10)	10 (6-16)	0.0046
SOFA score day 0 (*n *= 144)	10 (10-13)	12 (10-14)	0.721
PaO_2_/FiO_2 _day 0 (*n *= 125)	287 (112-370)	71 (60-99)	< 0.0001
Plasma lactate day 0 (mmol/l) (*n *= 131)	6.5 (3.1-10.8)	1.8 (1.2-3.1)	< 0.0001
RRT associated	61 (58%)	27 (51%)	0.402
Complications			
Bleeding plus surgery	34 (32.4%)	9 (17%)	0.057
Total of RBC units	14 (7-24)	9 (5-18)	0.0911
RBC units/day on ECMO	2 (1-3.3)	1 (0.6-1.7)	< 0.0001
Platelets	4 (1-8)	1 (0-3)	< 0.001
FFP	4 (1-11)	0 (0-4)	< 0.001
Cryoprecipitate	1 (0-2)	0 (0-1)	0.004
Neurological stroke	1 (0.9%)	1 (1.9%)	1
Vascular complications	10 (9.5%)	0 (0%)	0.017
At least one bloodstream infection	14 (13.3%)	7 (13.2%)	1

Data presented as n (%) categorical variables and median (interquartile range) for non-parametric variables. APACHE II, acute physiology and chronic health evaluation; Day 0, day of ECMO initiation; PaO_2_/FiO_2_, arterial partial pressure of oxygen to inspired oxygen fraction ratio; RRT, renal replacement therapy; RBC, red blood cells; SOFA, sequential organ failure assessment; ICU, intensive care unit; FFP, fresh frozen plasma.

### Complications

**Figure 2:**
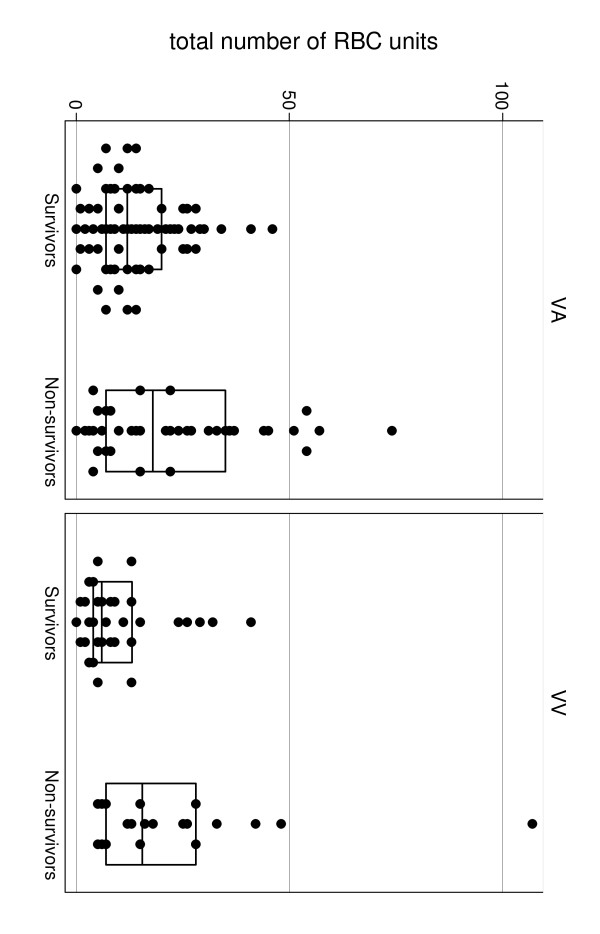
**Comparison of the number of red blood cell (RBC) units transfused during extracorporeal membrane oxygenation (ECMO) procedures between survivors and non-survivors in different types of ECMO**. VA ECMO, veno-arterial ECMO; VV ECMO, veno-venous ECMO.

Haemorrhagic complications were an issue for most of the patients on VA and VV ECMO and only four of them did not receive a RBC transfusion (one patient on VA ECMO post heart transplant was a Jehovah's Witness, a second had VA ECMO for acute myocardial ischaemia, and two others (one VV and one VA) were on ECMO for less than 48 hours). More than one-third of patients on VA ECMO required surgery for bleeding, while 17% of the patients on VV ECMO underwent surgery for bleeding issues. Surgical details on procedures to achieve haemostasis were not collected. The median number of RBC units transfused per day on ECMO was higher in patients who underwent VA ECMO than VV ECMO (Table 2) and was higher in patients who died regardless the types of ECMO (Tables 3, 4; Figure 2). Central VA ECMO was associated with a higher requirement of blood products than peripheral VA ECMO (17 RBC units (first and third quartiles: 12 to 26) versus 10 RBC units (first and third quartiles: 5 to 24), *P *= 0.005; and a median of 2.8 RBC units per day (first and third quartiles: 2 to 3.6) versus 2 RBC units per day (first and third quartiles: 1 to 3.3), *P *= 0.0018). Platelets, FFP and cryoprecipitate transfusion was also consistent, especially in the VA ECMO group, with only four patients who did not received platelets in the group (Table 2).

**Table 3:** Comparison between survivors and non-survivors among patients who underwent veno-arterial extracorporeal membrane oxygenation (VA ECMO).

Variables	Survivors (*n *= 66)	Non-survivors (*n *= 39)	OR, CI 95%	*P*
Age (years)	48 (42-58)	55 (44-62.5)	1.03 (1.00-1.06)	0.072
Male	52 (79%)	30 (77%)	0.90 (0.35-2.32)	0.823
APACHE II score (*n *= 102)	20 (15-25)	23.5 (17.8-28)	1.04 (0.99-1.10)	0.082
Comorbidities				
Chronic cardiac failure	39 (59%)	21 (54%)	0.81 (0.36-1.79)	0.6
Chronic respiratory failure	6 (9%)	3 (8%)	0.83 (0.20-3.54)	0.805
Immunosuppression	33 (50%)	19 (49%)	0.95 (0.43-2.10)	0.899
ECMO initiated in another hospital	11 (17%)	8 (21%)	1.29 (0.47-3.55)	0.621
Events before ECMO				
Pre-ECMO ICU days	0 (0-1)	0 (0-2.5)	1.07 (0.97-1.18)	0.165
Pre-MV ICU days	0 (0.0.75)	0 (0-1)	1.13 (0.97-1.31)	0.111
Cardiac arrest before ECMO	12 (18%)	12 (31%)	2.00 (0.79-5.04)	0.141
Subtype of VA ECMO			2.40 (1.03-5.60)	0.044
VA central (*n *= 43)	32/43(74%)	11/43 (25.5%)		
VA peripheral (*n *= 62)	34/62 (55%)	28/62 (45%)		
Post-operative ECMO	36 (56%)	18 (46%)	0.71 (0.32-1.58)	0.406
Severity at ECMO initiation				
SOFA score before ECMO (*n *= 90)	11 (10-13)	12 (11-14)	1.12 (0.97-1.29)	0.133
PaO_2_/FiO_2 _ratio before ECMO (*n *= 79)	297 (111-355)	279 (140-380)	1.00 (1.00-1.00)	0.955
Plasma lactate day 0 (*n *= 82) (mmol/l)	5.9 (3-8.4)	8.8 (3.6-13.1)	1.13 (1.02-1.25)	0.014
Other organ support				
RRT	34 (51%)	27 (69%)	2.12 (0.92-4.88)	0.078
Inotropes/vasopressors	60 (91%)	37 (95%)	1.85 (0.35-9.65)	0.465
Days on ECMO	7 (6-10)	8 (3.5-13)	1.05 (0.98-1.13)	0.197
ECMO complications				
Neurologic complications	0 (0%)	1 (2.6%)	-	-
Bloodstream infections	7 (11%)	7 (17.9%)	2.18 (0.72-6.56)	0.290
Bleeding plus surgery	20 (30%)	14 (36%)	1.29 (0.5- 2.98)	0.554
Number of RBC units				
Total	12 (7-20)	15 (7-34)	1.04 (1.01-1.07)	0.008
Per day on ECMO at the Alfred	2 (0.9-3.1)	2.3 (1.5-4)	1.11 (0.97-1.26)	0.115
Platelets (bag, 350 ml)	3 (1-6.25)	5 (0-12)	1.08 (1.01-1.15)	0.021
FFP (bag, 300 ml)	3.5 (0-8)	9 (2-15.5)	1.11 (1.04-1.18)	0.001
Cryoprecipitate (bag, 150 ml)	0 (0-2)	1 (0-3)	1.34 (1.05-1.71)	0.018
Vascular complications	4 (6%)	4 (10%)	1.77 (0.42-7.53)	0.438

Data presented as n (%) categorical variables and median (interquartile range) for non-parametric variables. APACHE II, acute physiology and chronic health evaluation; MV, mechanical ventilation; PaO_2_/FiO_2_, arterial partial pressure of oxygen to inspired oxygen fraction ratio; RRT, renal replacement therapy; RBC, red blood cells; SOFA, sequential organ failure assessment; ICU, intensive care unit; FFP, fresh frozen plasma.

**Table 4:** Comparison between survivors and non-survivors among patients who underwent veno-venous extracorporeal membrane oxygenation (VV ECMO).

Variables	Survivors (*n *= 35)	Non-survivors (*n *= 18)	OR, CI 95%	*P*
Age (years)	36 (25-49)	34 (25-50)	1.00 (0.96-1.04)	0.943
Male	18 (56%)	15 (75%)	2.50 (0.74-8.49)	0.142
APACHE II score (*n *= 50)	16 (13-20.5)	17 (13-21)	1.02 (0.92-1.12)	0.76
Comorbidities				
Chronic respiratory failure	8 (24%)	7 (35%)	1.68 (0.50-5.68)	0.402
Immunosuppression	10 (30%)	6 (30%)	0.99 (0.29-3.31)	0.981
Cystic fibrosis	3 (9%)	4 (20)	2.50 (0.50-12.57)	0.266
ECMO initiated in another hospital	14 (42%)	7 (21%)	0.73 (0.23-2.31)	0.593
Events before ECMO				
Pre-ECMO ICU days	2(0-4)	3 (0-7)	1.11 (0.99-1.24)	0.073
Pre-MV ICU days	1 (0-3)	1 (0-4)	1.08 (0.97-1.21)	0.177
Cardiac arrest before ECMO	2 (6%)	1 (5%)	0.82 (0.07-9.62)	0.872
Post-operative ECMO	4 (12%)	2 (10%)	0.81 (0.13-4.86)	0.813
Severity at ECMO initiation				
SOFA score before ECMO (*n *= 51)	11 (10-13)	13.5 (112-16)	1.20 (0.99-1.46)	0.064
PaO_2_/FiO_2 _ratio before ECMO (*n *= 43)	71 (68-99)	73 (60-91)	1.00 (0.97-1.03)	0.977
Plasma lactate (*n *= 46) (mmol/l)	1.6 (1.1-2.6)	2.9 (1.6-3.4)	1.08 (0.85-1.35)	0.532
Other organ support				
RRT	14 (42%)	13 (65%)	2.52 (0.80-7.95)	0.115
Inotropes/vasopressors	32 (97%)	19 (95%)	0.59 (0.04-10.05)	0.718
Days on ECMO	9 (6-12)	11.5 (6.8-22.5)	1.05 (0.99-1.11)	0.135
ECMO complications				
Neurologic complications	0 (0%)	1 (5%)	-	-
Bloodstream infections	6 (18%)	1 (5%)	0.24 (0.03-2.13)	0.199
Bleeding plus surgery	4 (12%)	5 (25%)	2.42 (0.56-10.35)	0.235
Number of RBC units				
Total	6 (4-13)	15.5 (7-28)	1.07 (1.013-1.13)	0.015
Per day on ECMO at the Alfred	0.75 (0.4-1.2)	1.7 (0.7-2.3)	1.82 (1.01-3.27)	0.047
Platelets (bag, 350 ml)	0 (0-2)	5 (0-7)	1.63 (1.21-2.21)	0.001
FFP (bag, 300 ml)	0 (0-4)	0.5 (0.5-5.3)	1.01 (0.93-1.10)	0.812
Cryoprecipitate (bag, 150 ml)	0 (0-0)	0 (0-1)	1.09 (0.85-1.41)	0.68
Vascular complications	0 (0%)	0 (0%)	-	-

Data presented as n (%) categorical variables and median (interquartile range) for non-parametric variables. APACHE II, acute physiology and chronic health evaluation; MV, mechanical ventilation; PaO_2_/FiO_2_, arterial partial pressure of oxygen to inspired oxygen fraction ratio; RRT, renal replacement therapy; RBC, red blood cells; SOFA, sequential organ failure assessment; ICU, intensive care unit; FFP, fresh frozen plasma.

Bloodstream infections ranked second behind haemorrhagic complications, in both types of ECMO. Of the 146 ECMO episodes that were continued for more than 48 hours, 24 bloodstream infections occurred during 21 ECMO episodes (14.4%). The median delay between ECMO initiation and bloodstream infection occurrence was 8 days (range 2 to 59 days) and the most frequent pathogens were Gram-negative bacilli including Enterobacteriaeceae, *Candida *sp. and *Enterococcus *sp. Neurologic complications of ECMO occurred in one patient in each ECMO type group. One patient, who was on heparin and had an international normalised ratio (INR) equal to 2, had a bilateral cerebral hemisphere haemorrhage, the other patient, who also was on heparin, had an intracerebral haematoma with intraventricular extension. Vascular complications occurred in eight patients, all who received VA ECMO (1/43 (2.3%) central and 7/62 (11.2%) peripheral, *P *= 0.14). The vascular complications were: a false aneurysm (one case), dissection of the femoral artery (one case), ischaemia secondary to delayed insertion of the distal perfusion cannula (two cases), venous thrombosis following surgical repair (one case) and data were not available for three cases. Two patients required a lower limb amputation because of ECMO. One had femoral vein injury and thrombosis following surgical repair of the artery at ECMO decannulation and developed venous infarction, the other did not have backflow cannula and developed arterial ischaemia.

### Patient outcomes

Of the 39 patients who died in the VA ECMO group (Table 5), mortality was attributed to the ECMO procedure in six (15%), to other treatment-related complications in 14 (36%) patients and to the primary illness in 19 cases (49%). The factors associated with increased risk of death in the univariate analysis are shown in Table 3. In the multivariate logistic regression analysis, the only factor independently associated with death was the number of RBC units (OR = 1.058, 95% CI 1.016 to 1.102, *P *= 0.007) (Table 6).

**Table 5:** Outcomes for veno-arterial extracorporeal membrane oxygenation **(**VA ECMO) and veno-venous extracorporeal membrane oxygenation **(**VV ECMO).

Variables	VA ECMO (*n *= 105)	VV ECMO (*n *= 53)	*P *value for VV versus VA ECMO
Primary outcomes			
-Hospital mortality	39 (37.1%)	20 (37.7%)	0.863
-Successful weaning	78 (74.3%)	37 (69.8%)	0.896

Secondary outcomes			
-ICU mortality	36 (34.3%)	18 (33.9%)	1
-ICU LOS after ECMO (days)	12 (4-20)	8 (0-15)	0.0373
-Hospital LOS after ECMO (days)	27 (12-48)	13 (0-33)	0.0063
-% of survivors transferred at home	37/67 (56%)	15/33 (45%)	0.40

Data presented as n (%) categorical variables and median (interquartile range) for non-parametric variables. ICU, intensive care unit; LOS, length of stay.

**Table 6:** Factors and complications associated with hospital mortality for each type of extracorporeal membrane oxygenation (ECMO) (multivariate analysis).

Variables	VA ECMO (*n *= 105) Odds ratio (95% CI), *P *value	VV ECMO (*n *= 53) Odds ratio (95% CI), *P *value
**Hospital mortality**		
Age	1.022 (0.983-1.063), 0.268	1.004 (0.947-1.073), 0791
APACHE II score	1.025 (0.963-1.092), 0.432	0.924 (0.793-1.076), 0.311
Plasma lactate day 0	1.114 (0.998-1.244), 0.053	-
Total of RBC units transfused	1.057 (1.016-1.102), 0.007	1.032 (0.959-1.109), 0.395
Peripheral VA ECMO	1.870 (0.610-5.729), 0.273	-
Number of platelets bags	-	1.572 (1.125-2.197), 0.008

VA ECMO, veno-arterial ECMO; VV ECMO, veno-venous ECMO; APACHE II, acute physiology and chronic health evaluation; RBC, red blood cell.

Of the 20 patients who died in the VV ECMO group (Table 5), mortality was attributed to the ECMO procedure in five (25%), to other treatment-related complications in three (15%) patient and to the primary illness in 12 cases (60%) (Table 5). In univariate analysis, the number of RBC units and platelets transfused were associated with mortality (Table 4). In multivariate analysis, only platelet transfusion remained significantly and independently associated with hospital mortality (OR = 1.458, 95% CI 1.087 to 1.955, *P *= 0.012) (Table 6).

Among the 27 VA ECMO episodes unsuccessfully weaned (25.7%), 16 involved patients who were never weaned from ECMO and 11 involved patients who underwent bridging procedures to transplantation. In univariate analysis, vascular complications were negatively associated with successful weaning, while post-operative VA ECMO had a higher chance to be successfully weaned (Additional file 1). Sixteen patients who underwent VV ECMO were never weaned; the volume of platelets transfused was predictive of unsuccessful weaning (Additional file 1).

Fifty-six percent and 45% of patients were discharged directly home in the VA and the VV ECMO group, respectively, while the others were transferred to another health service, including rehabilitation (Table 5).

### Changes over the study period

Trends over time were considered in two periods; from 2005 to 2007 when less than 20 ECMO procedures a year (a total of 59 ECMO) were performed, and from 2008 to 2011 when more than 20 ECMO episodes a year (a total of 99 ECMO) were performed. The proportion of VV ECMO trended to increase (25.4% of all the procedures during the first period versus 38.4% in the second period, *P *= 0.118). Peripheral ECMO became more frequent than central VA ECMO (22/44 during the first period versus 40/61 during the second period, *P *= 0.321). Between the first and the second periods, patient aged 44 (first and third quartiles: 30 to 58) years versus 48 (first and third quartiles: 36 to 57) years, *P *= 0.0869) and patient illness severity (APACHE II (19 (first and third quartiles: 14 to 23) versus 20 (first and third quartiles: 14 to 26), *P *= 0.292) and SOFA score (12 (first and third quartiles: 11 to 14) versus 11 (first and third quartiles: 10 to 14), *P *= 0.0661) were not significantly different. There was no difference in the number of RBC units transfused between the first study period (14 (first and third quartiles: 7 to 22) RBC units/episode or 1.8 (first and third quartiles: 0.9 to 3.3) RBC unit per day) and the second study period (12 (first and third quartiles: 5 to 25) RBC units/episode or 1.5 (first and third quartiles: 0.7 to 2.4) RBC unit per day). In addition, the occurrence of vascular (7/59 versus 5/99, *P *= 0.218), neurological (1/59 versus 1/99 versus, *P *= 1.0) complications and bloodstream infection (7/53, versus 17/93, *P *= 0.493) were similar between both study periods. There was also no statistical difference in mortality in the two time periods (40.68% versus 34.34%, *P *= 0.496).

## Discussion

We report the experience of a large Australian ECMO referral centre over a period of 5 1/2 years. For both types of ECMO, bleeding complications were the most frequent. For VA ECMO, bleeding complications, when expressed by the number of RBC units transfused during ECMO, was an independent factor of mortality. In patients who underwent VV ECMO, volume of platelets transfused was independently associated with hospital mortality.

Around a quarter of ECMO episodes required surgery for haemorrhage, which is similar to the 10 to 30% of haemorrhagic complication reported in the literature [28,29]. Surgery for haemorrhage was not associated with an increased risk of death while mortality increased with the number of RBC units transfused, suggesting that a readily identifiable and correctable site of bleeding was not as important as ongoing bleeding and transfusion. These results are comparable to previous publications and may also highlight the negative consequences of RBC transfusion in ICU patients [10,13,30,31]. Bleeding complications using alternative definitions have also been associated with poorer outcomes [2,13,24,32]. For instance, in a study in paediatric cardiac surgical patients, bleeding complication defined as 'uncontrolled mediastinal bleeding requiring surgical intervention' was independently predictive of death [24]. Bleeding events also estimated by gastrointestinal or pulmonary haemorrhage still affected the prognosis of 1,473 adults with ECMO [2]. Furthermore, we found that VA ECMO required more RBC units and other blood products than VV ECMO, possibly because half of VA ECMO was instituted in the operating room and intraoperative transfusions were included or because bleeding was a common feature of open chest central VA ECMO. In addition, transfusion policies in specific patient groups differ; for example, the transfusion threshold is higher in cardiac patients with uncorrected ischaemic heart disease. The association between the number of RBC units transfused and mortality in patients who underwent VA ECMO may be related to potential confounders including blood administered in the theatre for post-surgery ECMO. Interestingly transfusion of platelets was associated with a higher risk of death only for VV ECMO. The significance of this association remains uncertain. Platelet transfusion in VA ECMO may be only related to peri-operative haemorrhagic complications, while thrombocytopenia in patients on VV ECMO could be related either to coagulopathy that occurred on ECMO, or illness severity.

Infection ranked second among the complications, with bloodstream infections affecting 13% regardless of the type of ECMO. Our findings are in accordance with the literature reported where the percentage of patients who experienced bloodstream infections varied between 3.4% and 11.4% [14,17,19]. The impact of such infection on the outcome is controversial. Some authors highlight that sepsis and nosocomial infections acquired on ECMO increase the risk of death [13,14] whereas others do not report an independent relationship between infection and mortality [15,19]. Our findings support that bloodstream infections were not independently associated with an increased risk of death; however, our study may be underpowered to detect such association. Neurologic complications were rare in our series and may have been underestimated because of the difficulty in performing brain imaging procedures and the absence of systematic post mortem determination of intracranial pathology. Neurologic complications are highly variable in terms of incidence (between 4 and 37%). The majority consists of haemorrhage [3,21]. Vascular complications including leg amputation occurred exclusively in patients undergoing VA ECMO and in the same proportion as those reported by Wang *et al*. in a cohort of 62 VA ECMO for cardiogenic shock [33]. We found that vascular complications were associated with unsuccessful weaning in univariate analysis. In a cohort of 174 VV and VA ECMO episodes, vascular complications were not associated with mortality [34]. Larger studies are required to evaluate the impact of these complications on the chance of successful weaning.

We found that the lactate level before VA ECMO deployment was associated with poor outcome in univariate analysis but not in multivariate analysis (possibly because of a lack of power). Other studies have described plasma lactate 24 hours or 48 hours after ECMO initiation (as a marker of the adequacy of tissue perfusion) was a good predictor of death [13,35]. The absence of correlation between PaO_2_/FiO_2 _ratio prior to VV ECMO deployment and outcome was not surprising and in accordance with a recent report [2]. In our study, spontaneous cardiac arrest prior to ECMO initiation was not predictive of death. This result is in contrast to other studies, some of which included both before and after ECMO initiation [2,11,36]. Finally, for both types of ECMO, the duration of ECMO did not influence mortality in accordance with a previous report [12].

Although no change was significant between both periods of the study, ECMO patients in the second period tended to be older, have a higher severity of illness, whilst mortality tended to decrease.

### Strengths and limitations

Our study has several limitations. First, this is a single-centre retrospective study. Nonetheless, ECMO data and patients' characteristics were collected prospectively. Second, although this represents the totality of our experience over more than 5 years, the numbers of ECMO episodes was not high, precluding detailed subgroup analyses, which are likely to be underpowered for some outcomes. Although we did not collect data on haemoglobin levels prior to transfusion, previous work has suggested a high rate of concordance with the National Australian Transfusion Guidelines [37]. Furthermore, the study did not allow us to classify the bleeding complications and to explore the presence of coagulopathy or the anticoagulation characteristics. Similarly, information about oxygenator changes was not available and any correlation between oxygenator exchange and bleeding could not be done. We focused our infections analysis on bloodstream infections, because bloodstream infections have the highest impact on prognosis. Describing all infection types was not the aim of this study and has been already performed [23]. In addition, we were not able to establish correlation between infections and ECMO. Quality of life, really relevant outcome in this population, was not available and we were not able to evaluate long-term disability. Finally, our study did not include 'traditional' complications such as oxygenator failure, pump malfunction, air embolism, haemolysis or tubing rupture. Although current technology, staff training and simplified circuits have greatly reduced or completely removed the impact of these complications [9], the incidence of these complications was not recorded and therefore we were unable to assess their impact.

## Conclusions

In conclusion, ECMO implemented in a referral centre is a useful supportive therapy for temporary life-threatening cardiac and/or respiratory failure. Among the pre-ECMO parameters and the ECMO complications, the number of RBC units was independently associated with the mortality of patients on VA ECMO, while the volume of platelets transfused was associated with the risk of death in patients with VV ECMO. Our work supports the need to further explore prospectively the association between bleeding, blood products transfusion and mortality in patients with VV ECMO and VA ECMO.

## Key messages

• Bleeding is the most frequent issue in patients undergoing ECMO.

• Our study identified the volume of blood transfusion during ECMO, surrogate of bleeding, as an independent risk factor of mortality for VA ECMO.

• Platelet volume requirement in patients undergoing VV ECMO is an independent risk factor of death suggesting that coagulopathy in these patient group impacts on the outcome.

• Further studies identifying bleeding risk factors and coagulopathy management should occur to improve ECMO management and prognostic.

## Abbreviations

APACHE: acute physiology and chronic health evaluation; APTT: activated partial thromboplastin time; ECLS: extracorporeal life support; ECMO: extracorporeal membrane oxygenation; FFP: fresh frozen plasma; ICU: intensive care unit; INR: international normalised ratio; LOS: length of stay; MV: mechanical ventilation; PaO_2_/FiO_2_: arterial partial pressure of oxygen to inspired oxygen fraction ratio; RBC unit: red blood cell unit; RRT: renal replacement therapy; SOFA: sequential organ failure assessment; VA ECMO: veno-arterial ECMO; VV ECMO: veno-venous ECMO.

## Competing interests

The authors declare they have no competing interests.

## Authors' contributions

VP maintained prospectively the ECMO registry and DP maintained the ICU clinical database. CA collected clinical and biological data not available in the original databases and built a unique database. CA and AC performed the statistical analysis. CA, AC, DP and VP analysed the results. CA, AC, DP and VP wrote the manuscript. TL, CS, JC, GM helped to draft the manuscript. All authors revised and approved the final version of the manuscript.

## Supplementary Material

Additional file 1 **Variables associated with successful weaning from veno-venous extracorporeal membrane oxygenation (VV ECMO) and veno-arterial (VA) ECMO (univariate analysis)**. Click here for file
