# Supplementary material for: Factors associated with outcomes of patients on extracorporeal membrane oxygenation support: a 5-year cohort study
Source: Crit Care. 2013 Apr 18;17(2):R73. doi: 10.1186/cc12681 (PMC4056036; doi:10.1186/cc12681)
Supplement: Additional file 1 — Variables associated with successful weaning from veno-venous extracorporeal membrane oxygenation (VV ECMO) and veno-arterial (VA) ECMO (univariate analysis). [file cc12681-S1.DOCX]

**Additional file 1**

**Variables associated with successful weaning from VV ECMO and VA ECMO (univariate analysis)**

| **Variables** | **VA ECMO**  **OR, CI 95%** | **p** | **VV ECMO**  **OR CI 95%** | **p** |
| --- | --- | --- | --- | --- |
| Age, years | 1.011 (0.979-1.042) | 0.510 | 1.009 (0.967-1.054) | 0.655 |
| Gender, male | 0.757 (0.251-2.286) | 0.622 | 0.667 (0.192-2.314) | 0.523 |
| APACHE II score | 0.971 (0.922-1.023) | 0.273 | 1.018 (0.912-1.136) | 0.745 |
| Chronic respiratory failure | - | - | 0.535 (0.152-1.889) | 0.332 |
| Chronic cardiac failure | 1.091 (0.451-2.63) | 0.847 | - | - |
| Immunodepression | 1.983 (0.807-4.873) | 0.135 | - | - |
| ECMO initiated in another hospital | 0.519 (0.947-1.133) | 0.225 | 1.136 (0.340-.796) | 0.835 |
| Days on support | 1.036 (0.180-1.496) | 0.445 | 0.989 (0.934-1.048) | 0.719 |
| Pre ECMO ICU days | 0.931 (0.847-1.023) | 0.138 | 0.904 (0.814-1.005) | 0.062 |
| Pre ECMO ventilation days | 1.005 (0.881-1.147) | 0.936 | 0.907 (0.805-1.021) | 0.106 |
| SOFA score at ECMO initiation | 0.909 (0.776-1.065) | 0.239 | 0.829 (0.976-1.019) | 0.075 |
| RRT | 1.407 (0.584-3.396) | 0.446 | 0.737 (0.227-2.397) | 0.612 |
| PaO_2_/FiO_2_ ratio before ECMO | 1.004 (0.999-1.007) | 0.051 | 1.017 (0.985-1.049) | 0.295 |
| Cardiac arrest before ECMO | 0.6129 (0.227-1.653) | 0.334 | 0.857 (0.0721-10.189) | 0.903 |
| Post operative ECMO | 3.414 (1.331-8.751) | 0.011 | 2.343 (0.251-21.860) | 0.455 |
| Plasma lactate day 0 | 0.998 (0.899-1.106) | 0.964 | 0.936 (0.738-1.186) | 0.582 |
| RBC units transfused on ECMO | 0.995 (0.965-1.025) | 0.723 | 0.957 (0.914-1.00) | 0.063 |
| Platelets transfused on ECMO | 0.985 (0.922-1.052) | 0.657 | 0.794 (0.650-0.969) | 0.023 |
| Vascular complications | 0.176 (0.039-0.795) | 0.024 | - | - |
| Blood stream infection | 1.289 (0.457-3.634) | 0.631 | 1.931 (0.361-10.316) | 0.441 |

APACHE II: Acute physiology and chronic health evaluation; MV: mechanical ventilation; PaO_2_/FiO_2_: arterial partial pressure of oxygen to inspired oxygen fraction ratio; RRT: renal replacement therapy; RBC: red blood cells; SOFA: Sequential organ failure assessment; ICU: intensive care unit.
